# *N*-Methacryloyl-4-(piperidin-1-yl)-1,8-naphthalimide

**DOI:** 10.1107/S1600536810018994

**Published:** 2010-05-29

**Authors:** Lyall R. Hanton, Stephen C. Moratti, Zheng Shi, Jim Simpson

**Affiliations:** a Department of Chemistry, University of Otago, PO Box 56, Dunedin, New Zealand

## Abstract

In the title compound, C_21_H_20_N_2_O_3_, the naphthalimide unit is almost planar (r.m.s. deviation for the 15 non-H atoms = 0.059 Å). The carboximide N atom and the five C atoms of the 2-methyl­prop-2-enoyl substituent also lie in a plane (r.m.s. deviation = 0.009 Å), which subtends an angle of 84.34 (7)° to the naphthalamide plane. This orients the =CH_2_ group of the vinyl fragment towards the naphthalimide rings, giving the mol­ecule an extended configuration. The piperidine ring adopts a chair conformation and there is evidence for some delocalization between the naphthalene and piperidine units, the C—N_pip_ bond length being 1.404 (4) Å. In the crystal structure, π–π contacts with centroid–centroid distances of 3.5351 (18) and 3.7794 (18) Å supported by C—H⋯O hydrogen bonds link adjacent mol­ecules in a head-to-tail fashion, forming dimers. These are further stabilized by other C—H⋯O contacts of varying strength, which stack the mol­ecules down the *b* axis.

## Related literature

For background to the applications of 1,8-naphthalamides, see: McAdam *et al.* (2003 ▶, 2010 ▶); Flood *et al.* (2007 ▶). For their incorporation into polymer systems, see: Dana *et al.* (2007 ▶); Munro *et al.* (2008 ▶). For related structures, see: McAdam *et al.* (2003 ▶); Easton *et al.* (1992 ▶); Batchelor *et al.* (1997 ▶); Tagg *et al.* (2008 ▶). For comparative bond-length data, see: Allen *et al.* (1987 ▶) and for ring conformations, see: Cremer & Pople (1975 ▶).
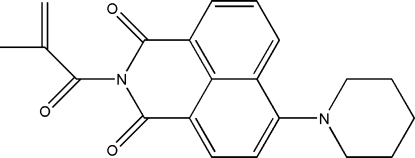


## Experimental

### 

#### Crystal data

C_21_H_20_N_2_O_3_
*M* *_r_* = 348.39
Monoclinic, 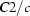

*a* = 29.049 (3) Å
*b* = 6.9852 (7) Å
*c* = 17.1503 (17) Å
β = 102.013 (6)°
*V* = 3403.7 (6) Å^3^
*Z* = 8
Mo *K*α radiation
μ = 0.09 mm^−1^
*T* = 90 K
0.65 × 0.11 × 0.04 mm

#### Data collection

Bruker APEXII CCD diffractometer
Absorption correction: multi-scan (*SADABS*; Bruker, 2006 ▶) *T*_min_ = 0.838, *T*_max_ = 1.000
12211 measured reflections
1843 independent reflections
1440 reflections with *I* > 2σ(*I*)
*R*_int_ = 0.075
θ_max_ = 21.2°

#### Refinement

*R*[*F* ^2^ > 2σ(*F* ^2^)] = 0.054
*wR*(*F* ^2^) = 0.152
*S* = 1.12
1843 reflections
236 parameters
H-atom parameters constrained
Δρ_max_ = 0.20 e Å^−3^
Δρ_min_ = −0.21 e Å^−3^

### 

Data collection: *APEX2* (Bruker, 2006 ▶); cell refinement: *APEX2* and *SAINT* (Bruker, 2006 ▶); data reduction: *SAINT*; program(s) used to solve structure: *SHELXS97* (Sheldrick, 2008 ▶); program(s) used to refine structure: *SHELXL97* (Sheldrick, 2008 ▶) and *TITAN2000* (Hunter & Simpson, 1999 ▶); molecular graphics: *SHELXTL* (Sheldrick, 2008 ▶) and *Mercury* (Macrae *et al.*, 2008 ▶); software used to prepare material for publication: *SHELXL97*, *enCIFer* (Allen *et al.*, 2004 ▶), *PLATON* (Spek, 2009 ▶) and *publCIF* (Westrip, 2010 ▶).

## Supplementary Material

Crystal structure: contains datablocks global, I. DOI: 10.1107/S1600536810018994/hb5457sup1.cif

Structure factors: contains datablocks I. DOI: 10.1107/S1600536810018994/hb5457Isup2.hkl

Additional supplementary materials:  crystallographic information; 3D view; checkCIF report

## Figures and Tables

**Table 1:** Hydrogen-bond geometry (Å, °)

*D*—H⋯*A*	*D*—H	H⋯*A*	*D*⋯*A*	*D*—H⋯*A*
C21—H21*B*⋯O12^i^	0.99	2.54	3.485 (4)	160
C24—H24*A*⋯O12^ii^	0.99	2.67	3.365 (4)	127
C21—H21*A*⋯O11^iii^	0.99	2.36	3.258 (4)	150
C25—H25*B*⋯O11^iv^	0.99	2.72	3.278 (4)	117

Symmetry codes: (i) 
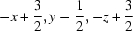
; (ii) 
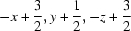
; (iii) 
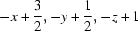
; (iv) 
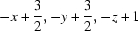
.

**Fig. 1.**
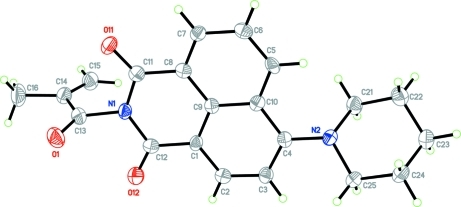
The structure of (I) showing displacement ellipsoids drawn at the 50% probability level.

**Fig. 2.**
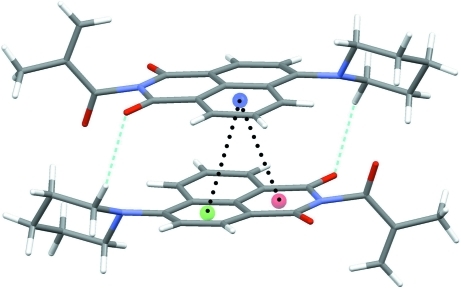
Head to tail dimers of (I) formed by π–π contacts (black dotted lines) augmented by C—-H···O hydrogen bonds (blue dashed lines). The blue sphere represents the centroid of the the C5···C8,C9,C10 ring, the red and green spheres those of the C1, C8, C9, C11, C12, N1 and C1···C4, C9, C10 rings respectively of an adjacent molecule. The symmetry operation linking the two molecules is 3/2-x, 3/2-y, 1-z.

**Fig. 3.**
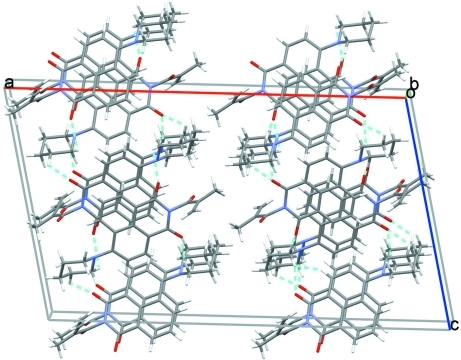
The crystal packing of (I) viewed down the b axis with hydrogen bonds drawn as blue dashed lines.

## supplementary crystallographic information

### Comment

We have recently been interested in naphthalimide derivatives as components of donor-acceptor arrays because, as good acceptors, they often exhibit strong fluorescence together with redox triggered LMCT transitions in the near-IR (McAdam *et al.* 2003, 2010; Flood *et al.* 2007). We have also incorporated fluorescent naphthalimides into polymer systems (Dana *et al.*, 2007; Munro *et al.*, 2008). Methacrylate derivatives are polymer precursors and the title compound, I, Fig 1, was synthesised to further scope the possibilities of incorporating fluorescent naphthalimide derivatives into polymers.

The title compound comprises a 1,8-naphthalimide ring system with a piperidino ring at the C4 position of the naphthalene ring and a 2-methyl-prop-2-en-1-one substituent on the N1 atom of the dicarboxamide ring. The naphthalimide unit is planar with an rms deviation from the best fit meanplane through all 15 non-hydrogen atoms of 0.0494 Å. The C13 atom of the propenone headgroup and the N2 atom of the piperidine ring are both displaced slightly from this plane with deviations 0.170 (4) and 0.004 (3) Å respectively both in the same direction. Bond lengths within the dicarboxamide ring are normal (Allen *et al.*, 1987) and consistent with a degree of delocalisation in the naphthalimide system. In keeping with previous observations (Easton *et al.*, 1992; Batchelor *et al.*, 1997; Tagg *et al.*, 2008) the N1—C13 bond is relatively long, 1.486 (4) Å, suggesting that there is a node at the N1 atom. In contrast the C4—N2 bond is short, 1.404 (4) Å, indicating a degree of delocalisation between the naphthalene and piperidine units. The piperidine ring adopts a classical chair conformation with Cremer-Pople puckering parameters [Q(2) = 0.005 (4) Å, φ(2) = 154 (5)° and Q(3) = -0.575 (43) Å (Cremer & Pople, 1975). The N1, C13, (O1), C14, C15, C16 segment of the propenone is also planar (rms deviation 0.0920 Å) and subtends an angle of 84.44 (7)° to the naphthalimide plane. This orients the =C15H_2_ of the vinyl fragment towards the naphthalimide rings.

In the crystal structure intermolecular π–π contacts occur between the unsubstituted C5···C8, C9, C10 naphthalene ring of one molecule and the C1, C8, C9, C11, C12, N1 carboxamide and substituted C1···C4, C9, C10 rings of an adjacent molecule to form head to tail dimers, with centroid to centroid distances of 3.5351 (18) and 3.7794 (18) Å respectively, Fig 2. These contacts are supported by weak C25–H25B···O11 hydrogen bonds. The dimers are further aggregated by a range of additional C—H···O contacts, Table 2, to give an extensive three dimensional network structure with molecules stacked down the *b* axis, Fig. 3.

### Experimental

4-Piperidine-1,8-naphthalic amide (200 mg, 0.72 mmol) was dissolved in distilled dichloromethane (DCM) (30 ml) under N_2_, triethylamine (3 ml) and methylacryoyl chloride (0.18 ml, 1.4 mmol) were added sequentially by syringe at 273 K. The mixture was stirred overnight. The mixture was then washed with distilled water (3 times) and the organic phase dried over MgSO_4_. The solvent was evaporated under reduced pressure and the crude product purified by silica gel column chromatography with hexane-ethyl acetate (50:50, v/v) to give an orange solid (168.3 mg, 0.48 mmol); yield of 67.2%. The compound was recrystallised by the slow diffusion of hexane into a concentrated solution of I in DCM to give very thin orange blades/needles of (I).

### Refinement

All H-atoms were refined using a riding model with d(C—H) = 0.95 Å, *U*_iso_=1.2*U*_eq_ (C) for aromatic, 0.99 Å, *U*_iso_=1.2*U*_eq_ (C) for CH_2_ and 0.98 Å, *U*_iso_ = 1.5*U*_eq_ (C) for CH_3_ H atoms. Crystals were very thin and weakly diffracting and measurable reflection data could not be observed beyond θ = 21.2 °. This results in both low data resolution and a poor data/parameter ratio.

### Crystal data

**Table:**

C_21_H_20_N_2_O_3_	*F*(000) = 1472
*M**_r_* = 348.39	*D*_x_ = 1.360 Mg m^−^^3^
Monoclinic, *C*2/*c*	Mo *K*α radiation, λ = 0.71073 Å
Hall symbol: -C 2yc	Cell parameters from 2032 reflections
*a* = 29.049 (3) Å	θ = 2.4–21.1°
*b* = 6.9852 (7) Å	µ = 0.09 mm^−^^1^
*c* = 17.1503 (17) Å	*T* = 90 K
β = 102.013 (6)°	Blade, orange
*V* = 3403.7 (6) Å^3^	0.65 × 0.11 × 0.04 mm
*Z* = 8	

### Data collection

**Table:**

Bruker APEXII CCD diffractometer	1843 independent reflections
Radiation source: fine-focus sealed tube	1440 reflections with *I* > 2σ(*I*)
graphite	*R*_int_ = 0.075
ω scans	θ_max_ = 21.2°, θ_min_ = 2.4°
Absorption correction: multi-scan (*SADABS*; Bruker, 2006)	*h* = −29→29
*T*_min_ = 0.838, *T*_max_ = 1.000	*k* = −7→6
12211 measured reflections	*l* = −17→17

### Refinement

**Table:**

Refinement on *F*^2^	Primary atom site location: structure-invariant direct methods
Least-squares matrix: full	Secondary atom site location: difference Fourier map
*R*[*F*^2^ > 2σ(*F*^2^)] = 0.054	Hydrogen site location: inferred from neighbouring sites
*wR*(*F*^2^) = 0.152	H-atom parameters constrained
*S* = 1.12	*w* = 1/[σ^2^(*F*_o_^2^) + (0.0822*P*)^2^] where *P* = (*F*_o_^2^ + 2*F*_c_^2^)/3
1843 reflections	(Δ/σ)_max_ < 0.001
236 parameters	Δρ_max_ = 0.20 e Å^−^^3^
0 restraints	Δρ_min_ = −0.21 e Å^−^^3^

### Special details

**Table:**

Geometry. All esds (except the esd in the dihedral angle between two l.s. planes) are estimated using the full covariance matrix. The cell esds are taken into account individually in the estimation of esds in distances, angles and torsion angles; correlations between esds in cell parameters are only used when they are defined by crystal symmetry. An approximate (isotropic) treatment of cell esds is used for estimating esds involving l.s. planes.
Refinement. Refinement of *F*^2^ against ALL reflections. The weighted *R*-factor wR and goodness of fit *S* are based on *F*^2^, conventional *R*-factors *R* are based on *F*, with *F* set to zero for negative *F*^2^. The threshold expression of *F*^2^ > σ(*F*^2^) is used only for calculating *R*-factors(gt) etc. and is not relevant to the choice of reflections for refinement. *R*-factors based on *F*^2^ are statistically about twice as large as those based on *F*, and *R*- factors based on ALL data will be even larger.

### Fractional atomic coordinates and isotropic or equivalent isotropic displacement parameters (Å^2^)

**Table:**

	*x*	*y*	*z*	*U*_iso_*/*U*_eq_	
C1	0.70398 (11)	0.5991 (4)	0.6119 (2)	0.0221 (9)	
C2	0.72082 (12)	0.6631 (5)	0.6879 (2)	0.0237 (9)	
H2	0.6991	0.7017	0.7193	0.028*	
C3	0.76855 (12)	0.6733 (5)	0.7204 (2)	0.0242 (9)	
H3	0.7788	0.7212	0.7730	0.029*	
C4	0.80161 (12)	0.6150 (4)	0.6777 (2)	0.0217 (9)	
C5	0.81694 (12)	0.5175 (4)	0.5443 (2)	0.0227 (9)	
H5	0.8499	0.5270	0.5640	0.027*	
C6	0.80025 (12)	0.4695 (4)	0.4661 (2)	0.0255 (9)	
H6	0.8219	0.4450	0.4326	0.031*	
C7	0.75190 (12)	0.4560 (4)	0.4347 (2)	0.0225 (9)	
H7	0.7408	0.4207	0.3805	0.027*	
C8	0.72057 (12)	0.4939 (4)	0.4825 (2)	0.0217 (9)	
C9	0.73615 (12)	0.5448 (4)	0.5643 (2)	0.0201 (9)	
C10	0.78588 (12)	0.5534 (4)	0.5964 (2)	0.0218 (9)	
C11	0.66975 (13)	0.4926 (4)	0.4468 (2)	0.0236 (9)	
C12	0.65327 (13)	0.5993 (5)	0.5773 (2)	0.0236 (9)	
C13	0.58901 (13)	0.5604 (5)	0.4598 (2)	0.0303 (10)	
C14	0.56012 (12)	0.3865 (5)	0.4482 (2)	0.0294 (10)	
C15	0.57626 (13)	0.2257 (5)	0.4834 (2)	0.0351 (10)	
H15A	0.6068	0.2217	0.5166	0.042*	
H15B	0.5574	0.1135	0.4757	0.042*	
C16	0.51218 (13)	0.4093 (6)	0.3979 (3)	0.0469 (12)	
H16A	0.4953	0.2871	0.3951	0.070*	
H16B	0.5147	0.4489	0.3441	0.070*	
H16C	0.4949	0.5068	0.4213	0.070*	
C21	0.87594 (12)	0.4410 (5)	0.7217 (2)	0.0300 (10)	
H21A	0.8647	0.3562	0.6754	0.036*	
H21B	0.8694	0.3770	0.7697	0.036*	
C22	0.92823 (12)	0.4714 (5)	0.7318 (2)	0.0346 (10)	
H22A	0.9352	0.5248	0.6821	0.041*	
H22B	0.9447	0.3471	0.7424	0.041*	
C23	0.94596 (13)	0.6084 (5)	0.8008 (2)	0.0380 (11)	
H23A	0.9424	0.5491	0.8516	0.046*	
H23B	0.9798	0.6360	0.8043	0.046*	
C24	0.91787 (12)	0.7929 (5)	0.7872 (2)	0.0350 (10)	
H24A	0.9281	0.8790	0.8334	0.042*	
H24B	0.9242	0.8580	0.7392	0.042*	
C25	0.86568 (12)	0.7558 (5)	0.7762 (2)	0.0300 (9)	
H25A	0.8588	0.6991	0.8254	0.036*	
H25B	0.8483	0.8781	0.7658	0.036*	
N1	0.63959 (9)	0.5398 (4)	0.49807 (17)	0.0237 (8)	
N2	0.85019 (10)	0.6250 (4)	0.70929 (16)	0.0262 (8)	
O1	0.57432 (8)	0.7182 (4)	0.43997 (15)	0.0387 (7)	
O11	0.65310 (8)	0.4540 (3)	0.37740 (14)	0.0293 (7)	
O12	0.62306 (9)	0.6481 (3)	0.61341 (15)	0.0331 (7)	

### Atomic displacement parameters (Å^2^)

**Table:**

	*U*^11^	*U*^22^	*U*^33^	*U*^12^	*U*^13^	*U*^23^
C1	0.020 (2)	0.018 (2)	0.026 (2)	−0.0025 (15)	−0.0009 (18)	−0.0004 (16)
C2	0.028 (2)	0.021 (2)	0.024 (2)	0.0006 (16)	0.0074 (19)	0.0028 (16)
C3	0.024 (2)	0.024 (2)	0.024 (2)	−0.0007 (16)	0.0020 (18)	0.0024 (16)
C4	0.021 (2)	0.0138 (19)	0.029 (2)	−0.0024 (15)	0.0029 (19)	0.0030 (15)
C5	0.021 (2)	0.0160 (19)	0.031 (2)	0.0053 (15)	0.0041 (18)	0.0035 (16)
C6	0.029 (2)	0.022 (2)	0.027 (2)	0.0027 (16)	0.0106 (19)	0.0023 (16)
C7	0.027 (2)	0.0163 (19)	0.022 (2)	0.0003 (15)	0.0016 (18)	−0.0002 (15)
C8	0.028 (2)	0.0070 (19)	0.029 (2)	0.0007 (15)	0.0031 (19)	0.0015 (15)
C9	0.021 (2)	0.0130 (19)	0.026 (2)	−0.0017 (15)	0.0034 (18)	0.0024 (16)
C10	0.028 (2)	0.0116 (18)	0.024 (2)	−0.0012 (15)	0.0005 (18)	0.0025 (15)
C11	0.029 (2)	0.016 (2)	0.025 (2)	0.0004 (16)	0.003 (2)	0.0036 (16)
C12	0.026 (2)	0.020 (2)	0.025 (2)	−0.0007 (17)	0.008 (2)	0.0005 (17)
C13	0.029 (2)	0.028 (3)	0.031 (2)	0.0066 (19)	0.0008 (19)	0.0009 (18)
C14	0.027 (2)	0.031 (3)	0.028 (2)	−0.0014 (19)	0.0020 (18)	−0.0012 (18)
C15	0.029 (2)	0.032 (3)	0.042 (3)	−0.0043 (19)	0.002 (2)	−0.003 (2)
C16	0.030 (2)	0.050 (3)	0.053 (3)	0.002 (2)	−0.011 (2)	−0.001 (2)
C21	0.027 (2)	0.024 (2)	0.036 (2)	0.0026 (16)	−0.0004 (19)	0.0035 (17)
C22	0.030 (2)	0.037 (2)	0.035 (2)	0.0060 (18)	0.0037 (19)	0.0006 (18)
C23	0.024 (2)	0.050 (3)	0.038 (3)	0.0006 (19)	0.0013 (19)	−0.008 (2)
C24	0.028 (2)	0.042 (3)	0.033 (2)	−0.0062 (18)	0.0034 (19)	−0.0139 (18)
C25	0.026 (2)	0.034 (2)	0.028 (2)	−0.0011 (17)	0.0019 (18)	−0.0093 (18)
N1	0.0192 (18)	0.0226 (17)	0.0269 (19)	−0.0013 (13)	−0.0011 (15)	−0.0011 (13)
N2	0.0242 (19)	0.0272 (18)	0.0248 (18)	0.0010 (14)	−0.0007 (14)	−0.0044 (14)
O1	0.0375 (17)	0.0292 (17)	0.0462 (18)	0.0089 (13)	0.0015 (14)	0.0043 (13)
O11	0.0325 (17)	0.0280 (15)	0.0238 (16)	−0.0017 (11)	−0.0024 (13)	−0.0014 (11)
O12	0.0269 (16)	0.0365 (16)	0.0349 (17)	−0.0011 (12)	0.0043 (14)	−0.0032 (12)

### Geometric parameters (Å, °)

**Table:**

C1—C2	1.368 (5)	C13—N1	1.486 (4)
C1—C9	1.415 (5)	C14—C15	1.315 (5)
C1—C12	1.469 (5)	C14—C16	1.486 (5)
C2—C3	1.384 (5)	C15—H15A	0.9500
C2—H2	0.9500	C15—H15B	0.9500
C3—C4	1.385 (5)	C16—H16A	0.9800
C3—H3	0.9500	C16—H16B	0.9800
C4—N2	1.404 (4)	C16—H16C	0.9800
C4—C10	1.440 (5)	C21—N2	1.480 (4)
C5—C6	1.370 (4)	C21—C22	1.508 (5)
C5—C10	1.418 (5)	C21—H21A	0.9900
C5—H5	0.9500	C21—H21B	0.9900
C6—C7	1.399 (5)	C22—C23	1.525 (5)
C6—H6	0.9500	C22—H22A	0.9900
C7—C8	1.372 (5)	C22—H22B	0.9900
C7—H7	0.9500	C23—C24	1.517 (5)
C8—C9	1.426 (5)	C23—H23A	0.9900
C8—C11	1.476 (5)	C23—H23B	0.9900
C9—C10	1.436 (5)	C24—C25	1.511 (5)
C11—O11	1.218 (4)	C24—H24A	0.9900
C11—N1	1.403 (4)	C24—H24B	0.9900
C12—O12	1.223 (4)	C25—N2	1.462 (4)
C12—N1	1.397 (4)	C25—H25A	0.9900
C13—O1	1.205 (4)	C25—H25B	0.9900
C13—C14	1.466 (5)		
			
C2—C1—C9	119.3 (3)	H15A—C15—H15B	120.0
C2—C1—C12	121.0 (3)	C14—C16—H16A	109.5
C9—C1—C12	119.6 (3)	C14—C16—H16B	109.5
C1—C2—C3	122.0 (3)	H16A—C16—H16B	109.5
C1—C2—H2	119.0	C14—C16—H16C	109.5
C3—C2—H2	119.0	H16A—C16—H16C	109.5
C2—C3—C4	121.2 (3)	H16B—C16—H16C	109.5
C2—C3—H3	119.4	N2—C21—C22	111.2 (3)
C4—C3—H3	119.4	N2—C21—H21A	109.4
C3—C4—N2	122.2 (3)	C22—C21—H21A	109.4
C3—C4—C10	119.0 (3)	N2—C21—H21B	109.4
N2—C4—C10	118.7 (3)	C22—C21—H21B	109.4
C6—C5—C10	121.2 (3)	H21A—C21—H21B	108.0
C6—C5—H5	119.4	C21—C22—C23	110.3 (3)
C10—C5—H5	119.4	C21—C22—H22A	109.6
C5—C6—C7	121.0 (3)	C23—C22—H22A	109.6
C5—C6—H6	119.5	C21—C22—H22B	109.6
C7—C6—H6	119.5	C23—C22—H22B	109.6
C8—C7—C6	119.7 (3)	H22A—C22—H22B	108.1
C8—C7—H7	120.2	C24—C23—C22	109.3 (3)
C6—C7—H7	120.2	C24—C23—H23A	109.8
C7—C8—C9	121.4 (3)	C22—C23—H23A	109.8
C7—C8—C11	118.8 (3)	C24—C23—H23B	109.8
C9—C8—C11	119.6 (3)	C22—C23—H23B	109.8
C1—C9—C8	121.5 (3)	H23A—C23—H23B	108.3
C1—C9—C10	120.0 (3)	C25—C24—C23	111.5 (3)
C8—C9—C10	118.4 (3)	C25—C24—H24A	109.3
C5—C10—C9	118.2 (3)	C23—C24—H24A	109.3
C5—C10—C4	123.2 (3)	C25—C24—H24B	109.3
C9—C10—C4	118.4 (3)	C23—C24—H24B	109.3
O11—C11—N1	119.4 (3)	H24A—C24—H24B	108.0
O11—C11—C8	124.5 (3)	N2—C25—C24	110.0 (3)
N1—C11—C8	116.1 (3)	N2—C25—H25A	109.7
O12—C12—N1	119.1 (3)	C24—C25—H25A	109.7
O12—C12—C1	124.1 (3)	N2—C25—H25B	109.7
N1—C12—C1	116.8 (3)	C24—C25—H25B	109.7
O1—C13—C14	124.1 (3)	H25A—C25—H25B	108.2
O1—C13—N1	118.2 (3)	C12—N1—C11	126.2 (3)
C14—C13—N1	117.7 (3)	C12—N1—C13	117.1 (3)
C15—C14—C13	120.4 (3)	C11—N1—C13	115.8 (3)
C15—C14—C16	124.0 (3)	C4—N2—C25	117.0 (3)
C13—C14—C16	115.5 (3)	C4—N2—C21	116.7 (2)
C14—C15—H15A	120.0	C25—N2—C21	111.5 (3)
C14—C15—H15B	120.0		

### Hydrogen-bond geometry (Å, °)

**Table:**

*D*—H···*A*	*D*—H	H···*A*	*D*···*A*	*D*—H···*A*
C21—H21B···O12^i^	0.99	2.54	3.485 (4)	160
C24—H24A···O12^ii^	0.99	2.67	3.365 (4)	127
C21—H21A···O11^iii^	0.99	2.36	3.258 (4)	150
C25—H25B···O11^iv^	0.99	2.72	3.278 (4)	117

Symmetry codes: (i) −*x*+3/2, *y*−1/2, −*z*+3/2; (ii) −*x*+3/2, *y*+1/2, −*z*+3/2; (iii) −*x*+3/2, −*y*+1/2, −*z*+1; (iv) −*x*+3/2, −*y*+3/2, −*z*+1.
